# Incremental healthcare cost burden in patients with atrial flutter only

**DOI:** 10.3389/fcvm.2023.1094316

**Published:** 2023-03-01

**Authors:** Kathryn M. Kim, Steven Y. Kim, Kathy L. Schulman, Michael H. Kim

**Affiliations:** ^1^Chicago Medical School, Rosalind Franklin University, North Chicago, IL, United States; ^2^School of Medicine, Des Moines University, Des Moines, IA, United States; ^3^Schulman Healthcare Consulting, New Bedford, MA, United States; ^4^CHI Health, School of Medicine, Creighton University, Omaha, NE, United States

**Keywords:** atrial flutter, healthcare costs, atrial fibrillation, sex differences, healthcare economics

## Abstract

**Background:**

Limited information is available on the costs related to atrial flutter only. This study provides a comprehensive estimate of the cost in patients with atrial flutter only versus matched patients without any atrial arrhythmia.

**Methods:**

Patients over 20 years of age with a minimum of one inpatient or two outpatient diagnosis codes for atrial flutter in 2005 and a minimum of 12 months of continuous enrollment pre- and post-index were identified using the MarketScan Commercial and Medicare databases. Atrial flutter patients were propensity matched to patients without atrial arrhythmias. Total costs for each patient for 12 months post-index were calculated. National cost was estimated using the projected prevalence of atrial flutter for 2010.

**Results:**

A total of 1,042 patients with atrial flutter only were successfully matched with comparison patients. For atrial flutter patients compared to matched controls without atrial arrhythmias, total mean annual cost per patient was 81% higher ($23,008 vs. $12,717) and mean annual inpatient expenditure was 214% higher ($8,518 vs. $2,713). When applied to national atrial flutter prevalence data, total incremental cost burden was estimated to be $687.9 million per year more than patients without atrial arrhythmias, primarily due to cardiovascular specific expenditure ($377 million, 55% of total) with 58% ($218.5 million) of the increased inpatient expenditure due to cardiovascular specific admissions and $159 million (23%) for atrial flutter specific care. Sex-related differences were also present in atrial flutter only patients.

**Conclusion:**

Although atrial flutter-only patients are less prevalent than atrial fibrillation patients, the national incremental cost burden in atrial flutter is substantial on a per-patient level.

## Introduction

Atrial fibrillation (AF) is the most prevalent sustained cardiac arrhythmia and may occur as AF only or concurrently with atrial flutter (1). Atrial Flutter (AFL) was originally described by Lewis et al. (2) and is a distinct clinical entity representing the second most common cardiac arrhythmia with approximately 200,000 new cases per year (3). The exact prevalence of AFL, in the absence of AF, is uncertain and has been estimated to be about two percent of those with sustained atrial arrhythmias (1).

The economic impact of AF is largely driven by hospitalizations and has been well described in prior studies (4–7). The incremental cost associated with AF in a 2011 study of both Medicare and Commercially insured patients was estimated at $8,705 which was 73% higher in AF patients than in propensity-matched controls. The authors extrapolated to 2010 AF prevalence with an estimated national cost burden in the United States between 6 and 26 billion dollars (8). A more recent study, focused on incident AF, primarily in commercially insured patients, suggests the incremental cost burden, unstandardized and unadjusted for inflation, may be as much as three times higher (9). In contrast, the literature on the economic burden in patients diagnosed with AFL only is sparse. This study presents data on AFL only patients that were acquired at the same time as the AF population (8) so that the two separate populations may be compared in 2010 cost estimations.

## Materials and methods

### Study design

This retrospective observational cohort study on the AFL only cohort was pre-specified and used methods reported by Kim et al. (8) in the study referenced in the introduction but applied to the subset of patients diagnosed with AFL in the absence of AF. The AFL only data represent a separate population distinct from the AF population reported in the 2011 study (8). These data were not included in the published 2011 study. In summary, health care data were collected from the MarketScan *Commercial Claims and Encounters* and *Medicare Supplemental and Coordination of Benefits* research databases for the period January 1, 2004 through December 31, 2006. These databases represented the health services of nearly 38 million employees, dependents, and retirees in the United States, with commercial, primary, or Medicare supplemental coverage through privately insured, employer- sponsored health plans (10). Both the Commercial and Medicare databases included individuals covered under a variety of fee-for-service, point of service, and capitated reimbursement schemes. Nearly half of US health plans contribute to these databases that represent a heterogenous patient population covered by a mix of payers. Thus, these data can be used in observational studies relevant to a “real-world” setting. The databases complied with all aspects of the Health Insurance Portability and Accountability Act of 1996 and the data were deidentified and thus exempt from Institutional Review Board approval.

### Patient identification

Patients, ≥20 years of age, with a minimum of one inpatient or two outpatient diagnoses of AFL, in any diagnosis position for non-diagnostic claims, on different days, less than 365 days apart [International Classification of Diseases, Ninth Revision, Clinical Modification (ICD-9-CM) diagnosis code 427.32] between January 1, 2005 and December 31, 2005, were eligible for inclusion. The first qualifying AFL diagnosis was designated the index diagnosis. Patients were required to have 12 months of continuous enrollment and complete data availability for the period immediately before and after the index diagnosis (other than in cases of inpatient death). Any diagnosis of AF, either in the 12 months pre or post-index, resulted in exclusion as did any claim based evidence of transient AF or hyperthyroidism.

Control patients, ≥20 years of age, without evidence of either AF or AFL (no diagnosis or receipt of antiarrhythmic drugs) between January 1, 2004 and December 31, 2006 were randomly selected from the database at a ratio of 200:1 to obtain a large pool of potential matches. Index dates were assigned to potential comparison patients to replicate the distribution of index dates for AFL only patients. Controls were required to have ≥12 months of continuous enrollment and complete data availability both pre- and post-index.

### Propensity score matching

Propensity score matching techniques documented to minimize the impact of selection bias in observational studies (11) were used to select control patients from the pool of comparison patients who shared similar demographic and clinical characteristics to the AFL only cohort. The methods employed were previously described in the 2011 study though it should be noted that in the current study, propensity score development and evaluation occurred separately for patients insured by Medicare versus a commercial insurer due to smaller numbers of patients in the AFL only cohort. Propensity scores were then computed using a logistic regression model, with the dependent variable comprising membership of the AFL only cohort and independent variables (covariates) including baseline demographics (age, sex, US Census Bureau geographic region, payer type, and index date) and cardiovascular comorbidities such as coronary artery disease, cardiomyopathies, mitral valve disease, other valvular disease, congestive heart failure, peripheral vascular disease, diabetes, hypertension, and other clinical conditions such as thyroid disease and pulmonary disease, as well as the Charlson-Deyo Comorbidity Index (12) and Chronic Disease Score (13). Each patient in the AFL cohort was matched with his/her closest control using a 1:1 “nearest neighbor matching” technique, with a caliper of 0.25 of the standard deviation of the estimated propensity score (14). The discriminative power of the propensity score model was evaluated using the area under the receiver operating characteristic curve, or *C*-statistic. Potential overfitting was avoided through use of a variance inflation factor and correlation matrix for all included variables (15). The success of propensity score matching was assessed by comparing the prematch and postmatch balance of identified covariates (11–14). A standardized difference between the 2 cohorts (mean difference expressed as a percentage of the average standard deviation of the variable’s distribution across the AFL and control cohorts) of <10% was considered indicative of good balance (16).

### Outcome measures

Direct costs in the current study employed methods identical to those presented in the 2011 AF study including methods used in the estimation of national cost burden (8). AFL-specific medical costs were defined as payments on claims for which the principal ICD-9-CM diagnosis code was 427.32. Healthcare expenditures were calculated for hospitalizations, emergency department visits, physician visits, laboratory testing, outpatient treatment, and outpatient prescriptions for a period extending 12 months post-index and reported in US$ at 2008 values, consistent with the original study. Costs were categorized into AFL-related costs (antiarrhythmic drugs and claims with a primary AFL diagnosis), other cardiovascular costs, and non-cardiovascular costs. Cardiovascular specific costs were identified using the ICD-9-CM diagnosis and procedural codes. Cardiovascular specific pharmaceutical costs were determined from the RedBook Cardiovascular Therapeutic Group 7 drug list.

Paired AFL and control patients were assigned to one of the 16 sex and age specific subgroups. The incremental cost of AFL per capita was calculated as the mean cost difference within each subgroup of paired AFL and control patients. Mean incremental costs were multiplied by the estimated national prevalence of AFL for the year 2010. The resulting costs were summed to estimate the total national cost burden of AFL for 2010.

The sensitivity of per-capita cost estimates to multivariable adjustment was evaluated using second-stage multivariable regression analysis (17). The variables used in calculating the propensity score were included in either an ordinary least squares regression model with log-transformed costs or a two-part model, where appropriate, to adjust for any small residual covariate imbalance between the two cohorts after propensity score matching.

### Statistical analysis

Data management and statistical analyses were conducted using STATA 9.2 (Stata Corp., College Station, TX, United States) for propensity score matching and SAS version 9.1.3 (SAS Institute Inc., Cary, NC, United States) for all other analyses. Student *t*-test and the Mantel-Haenszel test were used for comparison of continuous and categorical variables, respectively. A probability value of <0.05 was considered statistically significant.

## Results

### Study population

**FIGURE 1:**
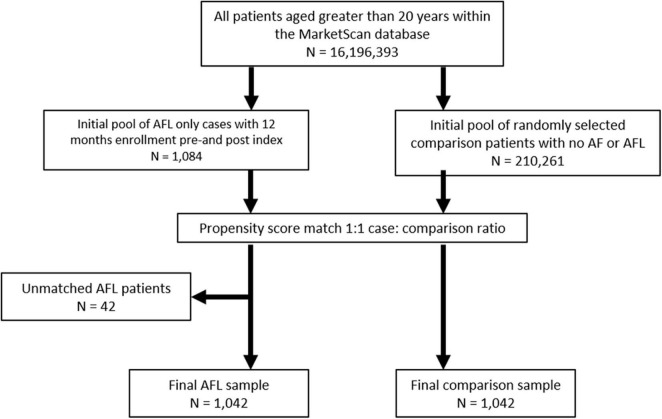
Patient identification algorithm.

From an initial pool of 1,084 AFL-only patients, 1,042 (96.1%) patients were successfully matched to an equal number of comparison patients (Figure 1). Propensity score models successfully matched 97% of the Medicare-insured AFL patients (*n* = 673) and 95% of the commercially-insured AFL patients (*n* = 369). The cohorts were well balanced (Table 1) after matching with standardized difference <10% on all covariates. The logistic regression models used to generate the propensity score showed good discriminative power (*C* = 0.85 and 0.87 in Medicare and Commercial models, respectively).

**TABLE 1:** Patient demographics and comorbidities (before and after matching).

	Prematch			Postmatch		
**Characteristics**	**AFL patient pool (*n* = 1,084)**	**Comparison patient pool (*n* = 210,261)**	Standardized difference (%)*	**AFL only patients (*n* = 1,042)**	**Medically matched control patients (*n* = 1,042)**	Standardized difference (%)*
**Sex, n (%)**						
Male	675 (62.3)	97,536 (46.4)	32.3	642 (61.6)	640 (61.4)	0.4
Female	409 (37.7)	112,725 (53.6)	-32.3	400 (38.4)	402 (38.6)	-0.4
Age, mean (SD)	67.4 (14.0)	46.3 (18.6)	128	67.3 (14.1)	68.0 (13.3)	-4.6
**Age category, y, n (%)**						
20–54	177 (16.3)	127,672 (60.7)	-102.5	176 (16.9)	166 (15.9)	2.6
55–59	107 (9.9)	24,395 (11.6)	-5.6	98 (9.4)	106 (10.2)	-2.6
60–64	129 (11.9)	15,891 (7.6)	14.7	119 (11.4)	110 (10.6)	2.8
65–69	127 (11.7)	9,698 (4.6)	26.2	127 (12.2)	112 (10.7)	4.5
70–74	140 (12.9)	8,165 (3.9)	33	135 (13.0)	160 (15.4)	-6.9
75–79	196 (18.1)	6,331 (3.0)	50.6	185 (17.8)	191 (18.3)	-1.5
80–84	132 (12.2)	3,735 (1.8)	41.7	128 (12.3)	119 (11.4)	2.7
≥85	76 (7.0)	2,057 (1.0)	31.2	74 (7.1)	78 (7.5)	-1.5
**Geographic region, n (%)**						
North Central	366 (33.8)	62,184 (29.6)	9	354 (34.0)	354 (34.0)	0
Northeast	94 (8.7)	22,024 (10.5)	-6.1	89 (8.5)	103 (9.9)	-4.6
South	328 (30.3)	79,798 (38.0)	-16.3	317 (30.4)	312 (29.9)	1
West	6 (0.6)	2,117 (1.0)	-5.2	276 (26.5)	262 (25.1)	3.1
Unknown	290 (26.8)	44,138 (21.0)	13.5	6 (0.6)	11 (1.1)	-5.3
**Payer type, n (%)**						
Commercial	389 (35.9)	176,943 (84.2%)	-113.2	369 (35.4)	369 (35.4)	0
Medicare	695 (64.1)	33,318 (15.8)	113.2	673 (64.6)	673 (64.6)	0
**Comorbidities, n (%)**						
Hypertension	836 (77.1)	52,108 (24.8%)	122.9	795 (76.3)	829 (79.6)	-7.9
Structural heart disease				516 (49.5)	470 (45.1)	8.9
Coronary artery disease	361 (33.3)	6,391 (3.0)	85.3	327 (31.4)	322 (30.9)	1
Mitral valve disease	123 (11.3)	1,697 (0.8)	45.2	104 (10.0)	99 (9.5)	1.6
Congestive heart failure	227 (20.9)	845 (0.4)	70.5	186 (17.9)	156 (15.0)	7.8
Cardiomyopathy	57 (5.3)	299 (0.1)	32	42 (4.0)	38 (3.6)	2
Diabetes	262 (24.2)	16,409 (7.8)	45.8	244 (23.4)	247 (23.7)	-0.7
Pulmonary disease	209 (19.3)	6,869 (3.3)	52.3	186 (17.9)	192 (18.4)	-1.5
Ischemic stroke	99 (9.1)	1,601 (0.8)	39.3	95 (9.1)	69 (6.6)	9.3
Peripheral vascular disease	61 (5.6)	1,183 (0.6)	29.6	55 (5.3)	49 (4.7)	2.6
Thyroid disease	62 (5.7)	4,080 (1.9)	19.8	56 (5.4)	60 (5.8)	-1.7
CCI, mean (SD)	1.6 (1.9)	0.3 (0.8)	87.4	1.5 (1.9)	1.5 (1.9)	-1
CDS, mean (SD)	6.8 (4.0)	2.3 (2.9)	129.3	6.6 (3.9)	6.8 (3.8)	-4.1

*A standardized difference <10% was considered indicative of a good balance in covariates between cohorts; AFL, indicates atrial flutter; CCI, Charlson-Deyo Comorbidity Index; SD, standard deviation; and CDS, Chronic Disease Score.

The postmatch study population was of mean age 67 and 68 years of age, respectively (62% of patients were ≥65 years), with a male:female balance of 62%:38%, drawn predominantly from the North Central and Southern regions of the United States as those two regions contributed greater numbers of arrhythmia patients to the database. The regional distribution of data reflect the underlying Marketscan data distribution. Medicare was the primary payer for most (65%) of the study population. Pre-existing AFL was present in 75% of patients and 16% showed no evidence of structural heart disease or hypertension at index. The mean Charlson-Deyo Comorbidity Index and Chronic Disease Score was 1.5 and 6.6, respectively. Approximately 74% of patients had some evidence of treatment for AFL during baseline, which was identified by receipt of any procedure classified as a non-pharmacologic treatment or use of drugs classified as antiarrhythmic, anticoagulant, or rate control. In particular, two-thirds of patients had evidence of a rate control prescription during baseline, 17% filled a prescription for an antiarrhythmic agent, and 16% had a non-pharmacologic intervention such as catheter ablation, pacemaker, and electrical cardioversion. About one quarter of AFL patients received warfarin. Direct Acting Oral Anticoagulants were not available until 2010 so were not included in the analysis. The mean duration of follow-up for the AFL and comparison patients was 359 and 365 days, respectively. The AFL cohort exhibited a higher rate of death within 12 months post-index (2.5 versus 0%).

### Annual direct costs per patient

**FIGURE 2:**
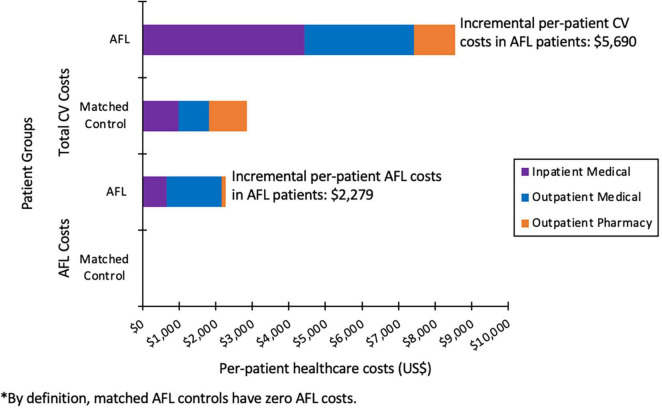
Total annual CV-specific and AFL-related healthcare costs per patient.

For AFL patients versus control patients not exhibiting any atrial arrhythmias, the total mean annual incremental cost per patient was $10,292 (81%) higher ($23,008 vs. $12,717, *P* < 0.001). The mean annual inpatient expenditure was $5,804 (214%) higher ($8,518 vs. $2,713, *P* < 0.001) for the AFL cohort over control patients, with a mean annual outpatient medical expenditure $4,301 (65.3%) higher ($10,880 vs. $6,579, *P* < 0.001). CV specific incremental costs were $5,690 (299%) greater in AFL patients versus matched control patients ($8,549 vs. $2,859, *P* < 0.001). This difference is primarily due to increased CV-specific inpatient cost, which was $3,449 (451.5%) higher ($4429 vs. $981, *P* < 0.001) in AFL patients versus control patients (Figure 2). Non-pharmacologic treatments comprised $3445 (15%) of the total per patient incremental cost burden vs. $6 for the control patients. Annual per patient AFL specific healthcare costs were $2,279, which comprised $653 for inpatient, $1,503 for outpatient medical, and $123 for outpatient prescription costs (Figure 2).

Sensitivity analysis indicated that propensity score matching addressed bias related to identified pre-index demographic and clinical differences between the two cohorts, although the potential for residual bias from unmeasured confounders remains. Second-stage multivariable adjustment had a minimal effect on cost estimates. The estimate of CV total expenditures fell by $1,525–$4,165 from the propensity-match based estimate of $5,690 while the estimate of CV inpatient expenditures fell by $429–$3,070 from the propensity-match based estimate of $3,449.

### National cost projection

Atrial flutter only cohort costs were projected, on an age-sex adjusted prevalence, to an estimated 74,466 annual cases based on US Census Bureau 2010 data. The total incremental cost burden associated with patients exhibiting AFL only was estimated to be $687.9 million per year over patients without atrial arrhythmias. Cardiovascular specific expenditure accounted for $376.7 million (54.8%) of the total cost burden with $219 million of the increased expenditure due to cardiovascular specific admissions. AFL specific care was found to account for $158.7 million (23%) of the total incremental cost burden of which 46 million was attributable to inpatient admissions with a primary diagnosis of AFL. Incremental non-cardiovascular costs were estimated to be $311.2 million, representing 45.2% of the total annual incremental cost of atrial flutter.

Men ($453 million) had a significantly higher national total incremental cost burden for all expenditures versus women ($253 million). Men had overall higher per patient incremental cost and national cost burden specific for treatment of AFL across the age groups with the exception of the 60–64 year age group. On a national and per patient cost level, AFL treatment specific costs decreased in men and women 75 years and older. Men also had a higher per patient incremental cost and national total cost burden ($291 versus $90 million) for CV specific expenditures, especially at ages <85. Mean total costs were greatest for men <55 years old at $25,643 per patient of which 75% of the value represented CV specific costs ($19,286). Per patient costs were more similar for men and women with AFL at 55 years and older. Data shows a trend of higher per patient inpatient costs for men whereas per patient outpatient costs were greater for women. National incremental inpatient cost for AFL was significantly higher in men in the majority of age categories.

## Discussion

Limited to no information is available regarding the healthcare cost burden of patients with AFL in the absence of AF on the US healthcare system. Findings from this investigation suggest that the incremental cost pattern in AFL patients is similar to, if not greater than, that observed in patients with AF when compared to matched controls with no atrial arrhythmias. Total healthcare costs were 81% higher for patients with AFL versus matched control patients without atrial arrhythmias ($23,008 vs. $12,717). Total incremental cost burden was 687.9 million dollars per year for AFL patients. Notably, there was a three-fold increase in cardiovascular-specific treatment costs. Cardiovascular inpatient spending was the major factor in the cost difference, being 4.5 times greater in AFL patients. AFL patients were three times more likely to have a cardiovascular specific hospitalization (16.5 versus 5.5%) with lengths of stay 50% longer when hospitalized (5 versus 3.3 days), and 4.7 times as likely to have a cardiovascular specific rehospitalization (2.7 versus 0.6%). The national cost projection for AFL-specific care was 159 million in 2010 dollars of which 29% (46 million) was attributable to inpatient admissions.

The AFL specific results of this study, based on a large, national, multiplayer data set provide comprehensive information on the incremental cost burden of AFL in the US. These data provide complementary data to the AF costs that are well recognized both on a population and per patient level. AFL only is a distinct clinical entity and thus information on such healthcare costs is of value. AFL patients have often been combined with AF patients despite distinct clinical and electrophysiologic differences as AF is substantially more common. An analysis of the Framingham Study data (18) showed that AFL, unlike AF, was not associated with valvular heart disease or hypertension, which was similar to the current investigation. The Framingham analysis included 112 AFL participants whom over the next 10 years had a significantly increased risk of developing AF (HR 5.01).

Current estimates indicate that over 3 million adults in the United States (US) have atrial fibrillation (AF) and 0.07 million adults in the US have atrial flutter (AFL). These figures are projected to increase to over 8 million for AF and over 0.5 million for AFL by 2050 (1). Patients with AF only, AFL only, and both AF and AFL have substantially greater burden of cardiovascular and cerebrovascular comorbidities than age- and gender-matched controls, notably coronary artery disease, congestive heart failure, valvular heart disease and stroke (1). There is evidence to suggest differences in clinical outcomes between AF and AFL. A recent study has indicated that AF and AFL are distinct in regards to comorbidities and prognosis for ischemic stroke, heart failure hospitalization, and all-cause mortality (19).

In the AFL only cohort, sex-related differences were present in healthcare utilization. AFL cost burdens were driven by men in the overall categories, including both on a per patient level and in national incremental costs. The highest cost burden was in younger patients less than 55 years old. Given the clear sex-related differences in resource utilization, potential disparities in care delivery may exist and further exploration as to why these findings were seen would need further study. Given the observational nature of these data, the exact reasons for these differences cannot be ascertained.

As with the original report of the any AF patients (8), the AFL only cohort report has the same limitations. These data are from a retrospective, non-randomized, observational study and despite being a pre-specified separate analysis, these data are now more than a decade older. Clinical practice has changed since these data were generated using 2010 projections, in particular the use of direct acting oral anticoagulants and a likely even greater use of ablation for first line treatment of typical AFL. Current costs compared to matched controls may be even higher than these older data. The age- and sex-specific national cost projections for AFL were based on mean values with fewer patients than in the AF study so the results should be interpreted with some caution. In addition, there is the potential for coarse AF to be misinterpreted and coded as AFL.

## Conclusion

This study indicates that direct medical costs in AFL only patients are significantly higher than in medically matched control subjects without atrial arrhythmias. AFL only costs were mainly driven by inpatient spending and non-pharmacologic treatments. Sex-related differences in AFL only healthcare utilization may indicate disparities in care delivery. Based on US age- and sex-specific prevalence data, the national incremental cost of AFL is estimated to be 687.9 million per year in 2010 dollars.

## Data availability statement

The datasets presented in this article are not readily available as the atrial flutter data were part of the original atrial fibrillation study in 2010. Requests regarding the data in the manuscript may be directed to the corresponding author.

## Ethics statement

Ethical review and approval was not required for the study of human participants in accordance with the local legislation and institutional requirements. Written informed consent from the patients was not required to participate in this study in accordance with the national legislation and the institutional requirements.

## Author contributions

KK and SK contributed to the data analysis, interpretation, presentations, and writing of the manuscript. KS contributed to the initial design, data acquisition and analysis, and writing of the manuscript. MK contributed to the initial design, data analysis, and writing of the manuscript. All authors have critically reviewed the contents and agree with its final contents and meet criteria for authorship.

## References

[1] Naccarelli G Varker H Lin J Schulman K. Increasing prevalence of atrial fibrillation and flutter in the United States. *Am J Cardiol.* (2009) 104:1534–9. 19932788 10.1016/j.amjcard.2009.07.022

[2] Lewis T Fell H Stroud W. Observations upon flutter and fibrillation: the nature of auricular flutter. *Heart.* (1920) 7:191–233.

[3] Granada J Uribe W Chyou PH Maassen K Vierkant R Smith PN Incidence and predictors of atrial flutter in the general population. *J Am Coll Cardiol.* (2000) 36:2242–6. 11127467 10.1016/s0735-1097(00)00982-7

[4] Wu E Birnbaum H Mareva M Tuttle E Castor A Jackman W Economic burden and co-morbidities of atrial fibrillation in a privately insured population. *Curr Med Res Opin.* (2005) 21:1693–9. 10.1185/030079905X65475 16238910

[5] Miyasaka Y Barnes M Gersh B Cha S Bailey K Seward J Changing trends of hospital utilization in patients after their first episode of atrial fibrillation. *Am J Cardiol.* (2008) 102:568–72. 10.1016/j.amjcard.2008.04.025 18721513 PMC3743254

[6] Coyne K Paramore C Grandy S Mercader M Reynolds M Zimetbaum P. Assessing the direct costs of treating nonvalvular atrial fibrillation in the United States. *Value Health.* (2006) 9:348–56. 16961553 10.1111/j.1524-4733.2006.00124.x

[7] Reynolds M Essebag V Zimetbaum P Cohen D. Healthcare resource utilization and costs associated with recurrent episodes of atrial fibrillation: the FRACTAL registry. *J Cardiovasc Electrophysiol.* (2007) 18:628–33. 10.1111/j.1540-8167.2007.00819.x 17451468 PMC1995078

[8] Kim M Johnston S Chu B Dalal M Schulman K. Estimation of total incremental health care costs in patients with atrial fibrillation in the United States. *Circ Cardiovasc Qual Outcomes.* (2011) 4:313–20. 21540439 10.1161/CIRCOUTCOMES.110.958165

[9] Deshmukh A Iglesias M Khanna R Beaulieu T. Healthcare utilization and costs associated with a diagnosis of incident atrial fibrillation. *Heart Rhythm O2.* (2022) 3:577–86. 10.1016/j.hroo.2022.07.010 36340482 PMC9626881

[10] Adamson D Chang S Hansen LG. *Health Research Data for the Real World: The MarketScan Databases*. New York, NY: Thomson Healthcare (2008). p. 1–32.

[11] Austin PC. A critical appraisal of propensity-score matching in the medical literature between 1996 and 2003. *Stat Med.* (2008) 27:2037–49. 10.1002/sim.3150 18038446

[12] Deyo R Cherkin D Ciol M. Adapting a clinical comorbidity index for use with ICD-9-CM administrative databases. *J Clin Epidemiol.* (1992) 45:613–9. 10.1016/0895-4356(92)90133-8 1607900

[13] Von Korff M Wagner E Saunders K. A chronic disease score from automated pharmacy data. *J Clin Epidemiol.* (1992) 45:197–203. 10.1016/0895-4356(92)90016-G 1573438

[14] Baser O. Too much ado about propensity score models? Comparing methods of propensity score matching. *Value Health.* (2006) 9:377–85. 10.1111/j.1524-4733.2006.00130.x 17076868

[15] Mansfield E Helms B. Detecting multicollinearity. *Am Stat.* (1982) 36:158–60. 10.2307/2683167

[16] D’Agostino R Jr. Propensity score methods for bias reduction in the comparison of treatment to a non-randomized control group. *Stat Med.* (1998) 17:2265–81. 10.1002/(SICI)1097-0258(19981015)17:19<2265::AID-SIM918>3.0.CO;2-B 9802183

[17] Stuart E. Matching methods for causal inference: a review and a look forward. *Stat Sci.* (2010) 25:1–21. 10.1214/09-STS313 20871802 PMC2943670

[18] Rahman F Wang N Yin X Ellinor P Lubitz S LeLorier P Atrial flutter: clinical risk factors and adverse outcomes in the Framingham heart study. *Heart Rhythm.* (2016) 13:233–40. 26226213 10.1016/j.hrthm.2015.07.031 PMC4698205

[19] Lin Y Chen T Ching C Lin M Tung T Liu C Different implications of heart failure, ischemic stroke, and mortality between nonvalvular atrial fibrillation and atrial flutter—a view from a national cohort study. *J Am Heart Assoc.* (2017) 6:e006406. 10.1161/JAHA.117.006406 28733435 PMC5586326

